# Contemporary Endothelial Genome Editing Technologies: Towards Precision Genetic Medicine for Vascular Diseases

**DOI:** 10.3390/ijms27115100

**Published:** 2026-06-04

**Authors:** You-Yang Zhao, Colin E. Evans

**Affiliations:** 1 Program for Lung and Vascular Biology, Section for Injury Repair and Regeneration Research, Stanley Manne Children’s Research Institute, Ann & Robert H. Lurie Children’s Hospital of Chicago, Chicago, IL 60611, USA; 2 Department of Pediatrics, Division of Critical Care, Northwestern University Feinberg School of Medicine, Chicago, IL 60611, USA; 3 Genetic Medicine and Nanotechnology Development Center (GeneMeNDer), Stanley Manne Children’s Research Institute, Ann & Robert H. Lurie Children’s Hospital of Chicago, Chicago, IL 60611, USA; 4 Departments of Pharmacology and Medicine, Northwestern University Feinberg School of Medicine, Chicago, IL 60611, USA; 5 Feinberg Cardiovascular and Renal Research Institute, Northwestern University Feinberg School of Medicine, Chicago, IL 60611, USA

**Keywords:** endothelial cell-specific gene knockout, genome editing, CRISPR, endothelium-targeted nanoparticle, cardiovascular biology, vascular diseases

## Abstract

Endothelial dysfunction is a key characteristic of many diseases, including atherosclerosis, hypertension, heart failure, stroke, cancer, acute respiratory distress syndrome (ARDS), peripheral vascular disease, coronavirus 2019 (COVID-19), and pulmonary arterial hypertension (PAH). To improve understanding of the roles of endothelial cells (ECs) in health and disease, EC-specific genome editing technologies have been developed in recent years. Therapeutic strategies that aim to restore a healthy endothelial monolayer include the inhibition of endothelial genes that cause EC injury and dysfunction and the induction or activation of endothelial genes that drive EC repair and regeneration. In this review, we describe established recombinase-mediated genetic modification technologies and emerging EC-specific genome editing technologies including viral and non-viral delivery of the CRISPR/Cas9 genome editing system, and we summarize the strengths and limitations of each technology. We then discuss possible avenues for future research, including the development of organ-specific EC genome editing technologies. In short, EC-specific genome editing technologies can be used to modulate gene expression selectively in ECs and even within a specific vascular bed and/or distinctive EC subtype, and, in doing so, greatly improve the understanding of vascular biology and help develop precision genetic medicine targeting the disease-causing vascular bed(s) to effectively treat diseases caused by vascular endothelial dysfunction.

## 1. Introduction

The vascular endothelium is a monolayer of endothelial cells (ECs) lining the luminal surface of all blood and lymphatic vessels. The endothelial monolayer is crucial for vascular homeostasis and maintenance of tissue fluid balance [1,2,3,4]. The endothelium also serves to maintain an anti-thrombotic and anti-inflammatory state in the vasculature and to control the proliferative and contractile state of underlying vascular smooth muscle cells [1,3,5,6,7]. When challenged by insults such as infection, tissue necrosis, immune reactions, oxidative/hemodynamic stress, or hypercholesterolemia, ECs become activated and/or damaged, which leads to inflammation and endothelial barrier disruption (i.e., increased vascular permeability, tissue edema, proinflammatory cytokine production, and leukocyte infiltration) [5]. Endothelial dysfunction is a key characteristic and driving force behind many diseases, including atherosclerosis, hypertension, heart failure, stroke, cancer and cancer metastasis, acute respiratory distress syndrome (ARDS), coronavirus 2019 (COVID-19), peripheral vascular disease, and pulmonary arterial hypertension (PAH) [6,8,9,10,11,12,13]. EC-specific gene modification technologies, including the traditional recombinase system such as the Cre/LoxP recombinase system, and emerging genome editing technologies, can act as fundamental experimental tools to improve understanding of EC biology in health and disease. Given that endothelial barrier integrity is a balance between EC injury and EC repair, EC-specific gene expression manipulations to inhibit EC injury and/or promote repair have been proposed as therapeutic strategies in the treatment of vascular diseases associated with EC dysfunction [14]. For instance, the downregulation of endothelial genes integral to EC leakage and death is one therapeutic strategy that could normalize the injured vasculature and improve endothelial integrity. Conversely, another therapeutic strategy that could restore a healthy EC monolayer is the upregulation of endothelial genes involved in endothelial regeneration and endothelial junction re-annealing [14,15].

Substantial advances in EC-specific gene expression manipulation methods have been made in the last 50 years. The aim of this review is to describe principles and examples of traditional genetic modification techniques and recent genome editing technologies that have been used to knockout (KO) genes selectively in ECs. Besides the classical but time-consuming Cre-*LoxP*-mediated KO technology, a recent genome editing technology has been established using the clustered regularly interspaced short palindromic repeats (CRISPR)-Cas9 system [16,17,18]. The CRISPR-Cas9 system has been leveraged for genome editing in various animal species, including mice and dogs [16,17,18,19,20], and will likely continue to materialize as a therapeutic approach in humans [21,22,23,24]. A recent study also shows the promise of direct genome editing in patients for treatment of genetic diseases [25]. In fact, the FDA has recently approved CRISPR-based therapies for sickle cell disease and beta thalassemia. Given the ability of the CRISPR-Cas9 system to correct disease-causing or associated mutations and to introduce gene KO, there is a substantial interest in the development of safe and efficient CRISPR-Cas9 delivery tools to target the vascular endothelium [19,20]. Despite understandable caution surrounding the progression of gene therapies into the clinic, novel technologies of EC-targeted genome editing could lead to the development of safe and effective treatments for a variety of vascular diseases that are associated with endothelial dysfunction.

## 2. Recombinase Systems of Endothelial Genetic Modification

**Figure 1:**
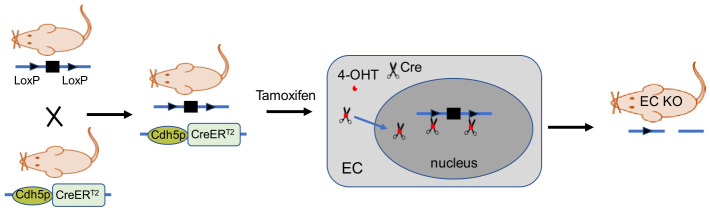
Inducible Cre-*LoxP*-mediated endothelial genetic modification. Conventional Cre-*LoxP*-mediated genetic modification in ECs is commonly generated by the crossbreeding of mice with the exon(s) of the gene of interest flanked by 2 *LoxP* sites in the intron regions, which normally do not disrupt gene expression, with mice that express the Cre recombinase driven by a EC-specific promotor (e.g., *Cdh5* promoter, *Cdh5p*). To generate inducible EC KO mice, the *Cre* mice express the fusion protein of Cre and mutated human estrogen receptor 1 (ER^T2^), which does not bind its natural ligand (17β-estradiol), but is activated by binding to the functional tamoxifen metabolite 4-hydroxytamoxifen (4-OHT). Tamoxifen-activated ER results in nuclear accumulation of the Cre-ER^T2^ fusion protein and thus Cre recombinase excises the *LoxP* sites in the chromosome for gene KO.

A common method for genetic modification in ECs is the Cre-*LoxP* recombinase system [21,22,23,24]*. LoxP* originated from bacteriophage P1 and consists of 34 base pairs containing two 13-base pair recognition sequences and an 8-base pair spacer/core sequence, which is palindromic (inverted repeat). The Cre recombinase specifically recognizes the *LoxP* sites, which are normally inserted into introns to flank the exon(s) for removal of the *LoxP*-flanked (floxed) exon(s) (Figure 1). Traditionally, this system is used to generate EC-specific KO mice in whom genes are deleted under the control of EC-specific promotors [25,26]. The number of Cre-driven mouse lines is growing, and this is evidenced by the creation of a database of Cre recombinase transgenic mice [27,28]. Among the growing list of EC-targeted Cre transgenic mice are *Apln-*Cre, *Bmx-*Cre, *Cdh5*-Cre, *Kdr-*Cre, *EndoSCL*-Cre, and *Tie2-*Cre [24,25,26,29,30]. *Cdh5*-Cre and *Tie2*-Cre are the 2 most commonly used Cre transgenic mouse lines for EC-restricted gene deletion. Given that some of the promoters such as *Tie2 and Cdh5* are also expressed in hematopoietic cells, especially during development, EC-specific KO mice have been generated by reconstituting wild type (WT) bone marrow cells into the mutant mice. Some studies have demonstrated important combinatory/synergistic roles of gene expression in hematopoietic cells and ECs, for example, *Tie2*-Cre-mediated disruption of *Egln1* (encoding prolyl hydroxylase 2, PHD2) in mice causes severe PAH due to the contribution of *Egln1* deletion in hematopoietic cells, while WT bone marrow transplantation leads to moderate PAH [31]. Another strategy to avoid non-EC deletion during development is to employ an inducible KO method (Figure 1). In the Cre-*LoxP* recombinase system, KO may be hereditary (i.e., present throughout development and life) or inducible (i.e., triggered from a selected time point) [32,33]. Inducible KO is commonly driven by the *Cre-ERT2* system, which relies upon the systemic administration of tamoxifen to drive gene deletion in *Cre*-expressing cells [32,34]. The tetracycline-inducible system has also been employed in some studies [35,36]. Although the *Cdh5*-CreERT2 transgenic is in general considered EC-specific, it has been shown that tamoxifen treatment at 2 weeks after birth also induces gene deletion in the endocardium. Sun et al. have generated EC-specific Cre driver mice, *Tg(Cdh5-rTA)D5Lbjn*, by subcloning the tetracycline-regulatable tTA after the *Cdh5* promoter [37]. Furthermore, the Cre-*LoxP* system can be used to remove the STOP signal leading to gene expression specifically in ECs, which is often used in genetic lineage tracing studies by breeding the EC-specific *Cre* mouse into the genetic background of a reporter mouse with eGFP or tdTomato expression under the control of the flox-STOP-flox cassette. Cre-mediated excision of the STOP signal will lead to EC-specific labeling.

Besides the Cre-*LoxP* system, there are other recombinase systems including Dre-*Rox* and Flp-*Frt* that can be employed for conditional gene modifications [38,39,40]. Owing to the limited mouse strains expressing Dre or Flp recombinase under control of EC-specific promoters, these two recombinase systems are not widely used for EC-specific genetic modification.

Limited by the length of time required to breed mouse strains with a floxed allele of the gene of interest with mouse strains expressing EC-specific Cre, the Cre-*LoxP*-mediated system of gene KO is not only time-consuming (1–2 years for breeding) but also expensive, as it requires the mouse strain with a floxed allele, and it is almost impossible to generate EC-specific gene KO in vertebrate animals other than mice owing to the challenge of genetic manipulation in large animal models. Cre toxicity is another caveat associated with tamoxifen-inducible and constitutive Cre-*LoxP*-mediated systems [41,42]. Due to the relative ease and robustness of the CRISPR/Cas9-mediated genome editing system, recent efforts have been dedicated to developing viral and non-viral delivery of the CRISPR/Cas9 system to ECs to induce genome editing selectively in ECs. Nevertheless, in our opinion, Cre-*LoxP*-mediated systems of EC genome editing remain a valuable tool for studies that aim to improve the understanding of EC-mediated mechanisms in vascular biology and disease. Future studies should aim to identify and utilize organ-specific EC Cre lines such as Tmem100 [43].

## 3. Viral Delivery of CRISPR/Cas9 System for Endothelial Genome Editing

Viral delivery systems include lentiviral, adenoviral, and adeno-associated viral systems. Song et al. used an adeno-associated virus (AAV) combined with the CRISPR/Cas9 system to target brain ECs and achieved an insertion/deletion (indel) efficacy of 36% in β-catenin (*Ctnnb1*) alleles; these authors then showed that the blood–brain barrier is impaired in *Tie2*-Cas9 *Ctnnb1* KO mice [44], partially resembling the phenotype of *EndoSCL*-CreERT2-mediated EC-specific *Ctnnb1* KO in adult mice [36]. KO efficiencies of ~40% and ~30% were observed in the levels of *Ctnnb1* mRNA and β-catenin protein, respectively [44]. The recombinant viral vector used in their study was brain microvascular EC-specific, which was a modified form of AAV serotype 2 [45].

Other investigators have used a recombinant AAV serotype 1-mediated CRISPR/Cas9 system to KO vascular endothelial growth factor receptor 2 (*Vegfr2*) in ECs, leading to decreased angiogenesis in murine models of oxygen-induced retinopathy and laser-induced neovascularization [46]. In this study, a dual-AAV vector system packaging guide RNA (gRNA) and Cas9 driven by the *Icam2*-specific promoter, which mainly targets vascular ECs was employed due to the packaging size limitation of the AAV vector. After intravitreal administration of the AAV to postnatal mice, a 2% indel efficiency was confirmed with next-generation sequencing, which resulted in a 30% reduction in VEGFR2 protein levels in the retina [46]. These studies have demonstrated a 30–40% protein KO efficiency. In a study of corneal EC genome editing, Uehara et al. used an intraocular injection of adenovirus encoding *Cas9* and gRNA for *Col8a2* to KO COL8A2 in corneal ECs [47]. This treatment resulted in approximately 24% indel efficiency in corneal ECs, reduced EC loss and restored corneal EC function in adult *Col8a2* mutant mice.

Different serotypes of AAV can be used to optimize transduction efficiency and limit immunogenicity [48]. However, the recombinant AAV system has several limitations, such as its low packaging capacity (~4.7 kb), the potential for an undesirable immune response, thus not suitable for repeated administration, and the possibility of DNA damage caused by viral vector-mediated extended expression of Cas9 [20,49].

So far, recombinant viral delivery of the CRISPR/Cas9 system has achieved relatively low indel efficiencies leading to insufficient gene KO in ECs in vivo. However, viral delivery of the CRISPR/Cas9-mediated genome editing in cultured ECs has been quite successful. Abrahimi et al. used lentiviral delivery of CRISPR-Cas9 in cultured primary human ECs and achieved biallelic gene disruptions in 40% of the cells [50]. Swiech et al. employed a dual-AAV vector system packaging Cas9 and gRNA [51]. Employing the EC-specific *Icam2* promoter to drive Cas9 expression, the recombinant AAV1-mediated CRISPR/Cas9 system induced an 80% KO of protein in cultured mouse primary brain microvascular ECs, as well as human primary retinal microvascular ECs (HRECs), and human primary umbilical vein ECs (HUVECs) [46,51].

Gong et al. co-delivered an adenovirus encoding Cas9 with a lentivirus encoding a *Tie2*-specific gRNA in primary human lung microvascular ECs to reduce the expression of the Tie2 [52]. Indel mutations were observed in 40–60% of *Tie2* sequencing reads and lasted for up to 5 passages with a 90% reduction in Tie2 protein [52]. Mutated ECs demonstrated increased EC permeability at baseline and following thrombin stimulation [52]. Gong et al. suggest that using gRNA only in the lentivirus, as opposed to both gRNA and Cas9, enabled a lower construct size and greater viral titer to be employed, thus improving transduction efficiency [52].

While viral delivery systems are amenable to high-throughput screening and circumvent the necessity for clonal expansion, there are several drawbacks, including limited packaging capacity and extensive generation protocols. In our opinion, viral EC gene editing represents a valid technique for studies that aim to improve the understanding and treatment of vascular diseases associated with EC dysfunction, but the limitations of this approach must be carefully considered. Future studies could aim to develop viral delivery systems to target all vascular ECs and/or enhance the genome editing efficiency.

## 4. Non-Viral Endothelial Genome Editing with the CRISPR/Cas9 System

### 4.1. Lipid Nanoparticle Delivery

Despite promising findings involving lipid nanoparticle-based genome editing, efficient genome editing with lipid nanoparticles can be a challenge for organs other than the eye and liver [53,54,55]. However, Cheng et al. recently showed that lipid nanoparticles with a specific formulation can be used for CRISPR/Cas9-mediated genome editing in ECs of a mouse lung [56]. Delivery of Cas9 mRNA and gRNA with SORT lipid nanoparticles comprising 50% DOTAP resulted in genome editing efficiency of 65% in ECs, 40% in epithelial cells, and 20% in immune cells in the lung. However, the protein KO efficiency was not defined, and there are no SORT lipid nanoparticles for the cardiovascular system yet.

Soni et al. employed cationic liposomes to deliver an all-in-one CRISPR plasmid DNA expressing Cas9 driven by the *CAG* promoter and Irak3-gRNA driven by the *U6* promoter to adult mice and achieved a 75% KO of IRAK-M protein levels in mouse lungs [57]. The cationic liposomes are made of dimethyldioctadecylammonium bromide/cholesterol liposomes [57,58,59]. It has been shown that these liposomes are trapped in the lung capillaries after intravenous administration due to their relatively large size; thus selectively target pulmonary vascular ECs but no other vascular beds [60].

### 4.2. Polymer Nanoparticle Delivery

**Figure 2:**
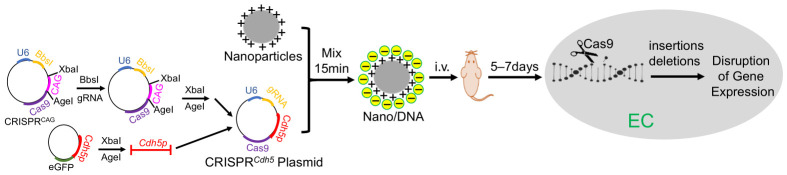
Endothelial genome editing by nanoparticle delivery of CRISPR/Cas9 plasmid DNA. Gene-specific gRNA is subcloned into the all-in-one CRISPR/Cas9*^CAG^* plasmid DNA expressing Cas9 under the control of *CAG* promoter (Addgene #48138) via restriction enzyme Bbs I digestion followed by gRNA oligonucleotide fragment ligation. Then, the *CAG* promoter is replaced with an EC-specific promoter (e.g., *Cdh5p*) via restriction enzyme digestion with Xba I and Age I followed by ligation with the *Cdh5p* fragment to generate the all-in-one CRISPR*^Cdh5P^* plasmid. EC-targeted polymer nanoparticles (e.g., EndoNP1) with positive charge are mixed with CRISPR*^Cdh5^* plasmid DNA for 10–20 min at room temperature, and then administered intravenously to mice. 5–7 days post-administration, CRISPR/Cas9-mediated genome editing [insertions and deletions (indel)] results in robust protein knockout in ECs. If 2 gRNAs specific for 2 different genes are expressed in the same plasmid, it will lead to generation of double knockout as simply as a single knockout. Depending on the promoter, it is easy to generate organ- and EC subtype-specific gene knockout.

To achieve EC-targeted delivery of nucleic acids, Zhang et al. generated poly(lactide-co-glycolide)-block-poly(ethylene glycol) methyl ether (PLGA-PEG) copolymer-based nanoparticles formulated with polyethyleneimine (PEI) (designated as EndoNP1) [19]. The EndoNP1 nanoparticles were mixed with a plasmid DNA expressing CRISPR/Cas9 driven by the *Cdh5* promoter and a gene-specific gRNA (e.g., *Pik3cg*, encoding the GPCR-dependent p110γPI3K) driven by the *U6* promoter at room temperature for 10–15 min, and then the nanoparticle/plasmid DNA mixture was administered retro-orbitally into adult WT mice. At 5–7 days after administration, tissues were collected for immunofluorescent staining and Western blotting to determine the protein KO efficiency [19] (Figure 2). This method led to approximately 80% protein KO, an efficiency similar to Cre-*LoxP* technology, in ECs of the lung, heart, aorta and peripheral vessels. It also induced marked genome editing in brain vascular ECs, although at a lower efficiency. In response to a lipopolysaccharide challenge, which induces endotoxemia leading to inflammatory vascular injury, CRISPR-mediated KO of endothelial p110γPI3K induced defective endothelial regeneration and vascular repair as seen in genetic p110γPI3K KO mice [19,59]. Employing a CAG promoter, which is active in all cell types, the authors showed that there is negligible genome editing in bone marrow cells, and a much lower rate (approximately one third of the efficiency seen in lung and heart ECs) in hepatocytes, demonstrating that the EndoNP1 nanoparticles predominantly target vascular ECs for nucleic acid delivery [19].

The authors further addressed whether there is a size limitation to the plasmid DNA that can be delivered by the EndoNP1 nanoparticles [19]. Employing a plasmid DNA of 12 kb expressing 2 gRNAs specific for 2 different genes (*Pick3cg* and *Vegfr2*) driven by the *U6* promoter and Cas9 driven by the *CDH5* promoter, it was shown that the 2 gRNA-plasmid system has the same efficiency as the single gRNA plasmid DNA system, and thus a mouse model with an EC-specific double KO can be generated in as little as 5–7 days.

Additionally, the authors tested another plasmid of 15 kb containing the CRISPR/Cas9 system and 2 additional transgenes (*eGFP* and *FOXM1*) under the control of the same *CDH5* promoter. Their data show the same genome editing efficiency with the simultaneous overexpression of FOXM1 in ECs, which rescued the defective endothelial regeneration and vascular repair phenotype induced by the CRISPR-mediated KO of endothelial p110γPI3K [19]. Together, these data have demonstrated that the EndoNP1 nanoparticle-mediated delivery of plasmid DNA after one i.v. administration can modify the expression of 1–4 genes simultaneously and selectively in ECs, including a single or double KO in as little as 5–7 days [19].

Employing this these-targeted EndoNP1 nanoparticles to deliver a CRISPR plasmid, others have also shown robust genome editing in ECs in young adult mice and aged mice [61,62,63]. These studies have shown that the polymer nanoparticle-mediated delivery of plasmid DNA is a useful tool to manipulate EC gene expression rapidly and efficiently and could be an effective approach for the genetic therapy of diseases caused by endothelial dysfunction, for example ARDS [19,63]. In a recent study by Huang et al., this nanoparticle-mediated gene delivery technology was used to deliver *FOXM1* to lung ECs of aged mice, leading to reactivated endothelial regeneration, normalized vascular repair, resolution of inflammation, and improved survival after a sepsis challenge [63]. As the nanoparticle/plasmid DNA mixture can be administered to mice at any age, including postnatal day 5 and 22–23 months [59,61,62,63,64], this technology can rapidly modify gene expression in ECs regardless of age and without the need for lengthy mouse breeding. The EndoNP1 delivery technique also has the same efficiency under basal conditions as it does under inflammatory injury conditions [26].

Yeh et al. also utilized PEI-based nanoparticles integrated with CRISPR-Cas9-mediated genome editing, with Cas9 driven by the *CDH5* promotor and two gRNAs targeting 2 *Txndc5* introns driven by the *U6* promoter [65]. In their study, nanoparticles were generated by complexing cationic polymers, such as PEI, with negatively charged plasmids, leading to an approximately 70% reduction in mRNA levels in ECs; however, they did not provide data demonstrating the knockout efficiency of the protein levels [65]. In *ApoE* KO mice, EC-specific deletion of *Txndc5* reduced atherosclerosis and vessel wall thickening [65]. Another study by Huang et al. employed the same PEI polymer to deliver a Klf2 plasmid to mice and showed a 2–3-fold increase in Klf2 mRNA expression in mouse lungs at 30 h after i.v. administration. This study suggests a relatively poor efficiency in in vivo delivery [66].

In our opinion, non-viral endothelial genome editing, including via EC-targeted nanoparticle systems, represents an emerging but important technique for the study of EC biology and vascular diseases. Future studies should aim to test these technologies in large animal models including non-human primates, and eventually in clinical trials.

## 5. Future Directions for Endothelial Genome Editing

### 5.1. EC Subtype- and Organ-Specific Genome Editing

Endothelial heterogeneity is a key characteristic feature of ECs [67,68,69]. ECs from different vascular beds are different, and there may be different subtypes of ECs in each vascular bed. Recent single-cell RNA sequencing analysis has revealed that there are pulmonary arterial ECs, venous ECs, general capillary ECs (gCAPs) and aerocytes (aCAPs), as well as lymphatic ECs in the lung [70]. Given the increasing appreciation of EC heterogeneity in animal models and humans, new methods to target EC sub-populations or specific organs will provide appealing research tools for the assessment of EC function in different vascular beds and EC subtypes. More importantly, such techniques will lead to precision genetic therapy by precisely modifying gene expression in ECs of the disease-causing vascular bed, such as selectively in pulmonary vascular ECs for PAH through the EC-targeted nanoparticle delivery of a gene or CRISPR system driven by the organ- and EC (subtype)-specific promoter(s). Future studies are warranted to identify novel organ- and EC-specific promoters or EC subtype-specific promoters. Recent singe-cell RNA sequencing [71] and whole-RNA sequencing [72] analyses of different organs have provided rich resources for the identification of EC genes unique to each vascular bed. Liu et al., employing single-cell RNA sequencing data and *Tmem100-*Cre-ERT2 mice, provided strong evidence that transmembrane protein 100 (*Tmem100*) is a gene specific to lung ECs [43]. Given the expression of Tmem100 in atrium myocytes and some pericytes and smooth muscle cells, Huang et al. further developed a dual promoter system driven by both the *CDH5* and *Tmem100* promoters to selectively target pulmonary vascular ECs [63]. Future studies could aim to discover other unique promoter systems that are specific to an EC subtype in a specific organ. EC-targeted nanoparticles will facilitate the validation of these unique promoter systems and have great potential for precision genetic medicine in human diseases by precisely targeting the disease-causing EC subtype(s) in a specific organ.

### 5.2. Genome Editing by EC-Targeted Nanoparticle Delivery of the CRISPR System in Rats and Large Mammals

The PLGA-PEG-based cationic polymer nanoparticle (EndoNP1) [26] is highly efficient in delivering the CRISPR system to mice at any age for robust endothelial genome editing in various organs. It is currently unknown whether this technology works as successfully in rats and large animals as it does in mice. If successful, this tool could enable the genetic modification of gene expression in the vascular ECs of rats and large mammals such as pig and non-human primates. More importantly, many of the “undruggable” targets can be easily manipulated by this EC-targeted nanoparticle delivery technology for the treatment of human diseases.

### 5.3. Development of Powerful Genome Editor(s) with High Fidelity

There is considerable and warranted concern regarding the potential off-target effects of the CRISPR/Cas9 system. With the identification of Cas9 mutants with higher fidelity, such as eCas9 [73], Cas9-HF1 [74], and hyproCas9 [75], the off-target effects can be substantially reduced. The development of the prime editing system further offsets the concerns regarding double-strand DNA breaks of the CRISPR/Cas9 system [76]. Future efforts should address the in vivo efficiency of the prime editing system in inducing genome editing in ECs as it shows only an 11% KO efficiency in retinal vascular ECs [77] or in the heart [78]. Future efforts should also aim to develop new or modified genome editor system(s) with minimal or virtually no off-target effects for therapeutic genome editing to treat vascular diseases.

### 5.4. Highly Efficient Non-Viral Delivery Systems for Cultured ECs

Zhang et al. developed an effective EC-targeted nanoparticle-based CRISPR/Cas9 system for robust genome editing in ECs in mice with an efficacy similar to Cre-*LoxP* system-mediated gene KO [19]. However, there is no evidence to determine whether this non-viral technology works well in cultured ECs. Previous studies have shown that the dual recombinant adenoviral and lentiviral system is efficient in delivering the CRISPR system to cultured ECs [52]. The generation of this system is quite tedious, however, and the viral system may cause unwanted side effects, including EC activation and long-term Cas9 expression and its associated off-target effects. Future efforts are warranted to develop effective non-viral nanoparticles for in vitro delivery of the CRISPR system or other genome editors with ≥80% genome editing efficiency in cultured ECs.

## 6. Conclusions

EC-specific genome editing technologies are valuable tools to improve the understanding of the role of EC biology in health and diseases. The Cre-*LoxP* technology and the Dre-*Rox* technology, as well as the Flp-*Frt* technology are precise genome engineering tools to manipulate genomic DNA in a specific tissue and/or at specific times. Their major advantages are precise and tissue- or cell-specific editing, providing cleaner results than a null mutation (global knockout). These technologies are the cornerstone for modifying gene expression in a specific tissue and/or cell type(s) in mice, which greatly facilitates biomedical research and the understanding of the molecular mechanisms of cardiovascular system development and cardiovascular diseases. However, this genetic technology cannot be directly applies to large mammals and humans and thus has limited translational value. Some efforts have been devoted to developing the viral delivery of genome editors for endothelial genome editing in animal models. Owing to the poor targeting of vascular ECs in vivo, viral delivery technologies have limited value in cardiovascular research. The recent development of EC-targeted nanoparticles for the delivery of the CRISPR system or other genome editors has provided unprecedented ease, speed, and robustness to modulate the expression of a single gene or multiple genes simultaneously in ECs in various organs, including the heart, lung, aorta, peripheral vessels and brains, and more importantly, in a specific vascular bed or EC subtype involved in disease development and progression (Table 1). Simple and efficient approaches for EC-specific in vivo genome editing at any age will provide a powerful research tool to quickly delineate gene function in the vascular system and to facilitate our understanding of the roles of ECs in health and disease. These advanced EC-specific genome editing technologies have the potential to become effective precision genetic medicines by targeting the specific disease-causing vascular bed(s) for diseases characterized by endothelial dysfunction, such as atherosclerosis, hypertension, stroke, acute myocardial function, ARDS, pulmonary arterial hypertension, and cancer.

## Figures and Tables

**Table 1:** Strengths and Weaknesses of Endothelial Gene Expression Modification Technologies.

Method	Strengths	Weaknesses	Refs.
Genetic knockout (KO) (mainly via Cre-*LoxP*, or Cre-ER^T2^-*LoxP*)	High fidelity Some Cre mouse lines are available Spatial tracking studies are possible using reporter mice (e.g., *GFP*, *tdTomato,* etc.) to enable visualization of Cre-positive ECs Ability to target all ECs or some EC sub-populations Cre-ER^T2^ can be used to temporally induce EC KO from a selected time EC genetic modification is permanent and can be transferred to offspring	Laborious and expensive to generate a *Cre* line and a mouse strain with a floxed allele of a gene of interest Breeding of mice to reach experimental age can take many months It is expensive and time-consuming to generate double KO mice or mice with multiple gene modifications KO efficiencies can be inconsistent (e.g., due to generational drift and variation in copy number) Cre-mediated toxicity can lead to growth inhibition, DNA damage, and activation of the immune response Cre expression is generally evident from embryonic stages of development and may trigger genetic compensation	[79,80,81]
Viral delivery of CRISPR for genome editing (e.g., via recombinant lentivirus, adenovirus, and adeno-associated viruses)	Relatively quick to generate CRISPR/Cas9-mediated genome editing Postnatal modification without genetic compensation Amenable to high-throughput screening	EC infection is not uniform and thus poor genome editing efficiency in general Sequestration by the liver and elimination by the immune system Potential for off-target effects and episomal degradation Possibility of DNA damage and activation of the immune response Cannot be administered repeatedly Laborious to generate and amplify recombinant viruses AAV has limited packaging capacity (~4.7 kb) Genome editing cannot be passed through the offspring	[44,45,46]
Non-viral delivery of CRISPR for genome editing (e.g., via Endo-NP1 nanoparticles)	Relatively simple procedure using plasmid DNA, with no size limitation Rapid (as little as 5 days) Robust genome editing efficiency (as high as Cre-*LoxP* technology) Ability to simultaneously edit multiple genes Ability to target all ECs or EC sub-populations Compatible with repeated dosing Can be used at any age after birth without developmental genetic compensation Compatible for RNA delivery (any nucleic acids)	Potential off-target effects associated with CRISPR-mediated genome editing Some degree of mosaic gene knockout Although genome editing is permanent, it cannot be passed through the offspring	[19,63,82]

## Data Availability

No new data were created or analyzed in this study. Data sharing is not applicable to this article.
